# Telemedicine in diabetes foot care delivery: health care professionals’ experience

**DOI:** 10.1186/s12913-016-1377-7

**Published:** 2016-04-18

**Authors:** Beate-Christin Hope Kolltveit, Eva Gjengedal, Marit Graue, Marjolein M. Iversen, Sally Thorne, Marit Kirkevold

**Affiliations:** Faculty of Health and Social Science, Centre for Evidence-Based Practice, Bergen University College, Bergen, Norway; Department of Global Public Health and Primary Care, University of Bergen, Bergen, Norway; Faculty of Health and Social Care, Molde University College, Molde, Norway; Departement of Medicine, Section of Endocrinology, Stavanger University Hospital, Stavanger, Norway; School of Nursing, University of British Columbia, Vancouver, Canada; Department of Nursing Science, Institute of Health and Society, University of Oslo, Oslo, Norway

**Keywords:** Telemedicine, Diabetic foot ulcer, Focus groups, Interpretive Description, Health care professionals

## Abstract

**Background:**

Introducing new technology in health care is inevitably a challenge. More knowledge is needed to better plan future telemedicine interventions. Our aim was therefore to explore health care professionals’ experience in the initial phase of introducing telemedicine technology in caring for people with diabetic foot ulcers.

**Methods:**

Our methodological strategy was Interpretive Description. Data were collected between 2014 and 2015 using focus groups (*n* = 10). Participants from home-based care, primary care and outpatient hospital clinics were recruited from the intervention arm of an ongoing cluster randomized controlled trial (RCT) (Clinicaltrials.gov: NCT01710774). Most were nurses (*n* = 29), but the sample also included one nurse assistant, podiatrists (*n* = 2) and physicians (*n* = 2).

**Results:**

The participants reported experiencing meaningful changes to their practice arising from telemedicine, especially associated with increased wound assessment knowledge and skills and improved documentation quality. They also experienced more streamlined communication between primary health care and specialist health care. Despite obstacles associated with finding the documentation process time consuming, the participants’ attitudes to telemedicine were overwhelmingly positive and their general enthusiasm for the innovation was high.

**Conclusions:**

Our findings indicate that using a telemedicine intervention enabled the participating health care professionals to approach their patients with diabetic foot ulcer with more knowledge, better wound assessment skills and heightened confidence. Furthermore, it streamlined the communication between health care levels and helped seeing the patients in a more holistic way.

## Background

In recent years, telemedicine (also referred to as telehealth, telemedicine and telecare) has been introduced as a potential method for delivering follow-up care to people with diabetic foot ulcers [1–3]. Although a handful of small quantitative studies [4, 5] have suggested that telemedicine is a feasible method, randomised controlled trials are needed to determine ways to deliver diabetes foot care that are contextually appropriate, feasible and sustainable among people with diabetic foot ulcers [6]. However, where complex interventions are concerned, the evidence from RCTs can be incomplete without qualitative approaches to capture ongoing processes and provide more indepth insight into the experiences of health care professionals when new technology is introduced.

The study we describe in this paper is a part of a larger cluster randomized controlled trial designed to investigate telemedicine follow-up care for people with diabetic foot ulcers in primary health care in collaboration with specialist health care. The intervention consists of an interactive telemedicine platform with a web based ulcer record combined with images sent by mobile phone from primary health care to specialist health care for wound assessment. What has been learned from prior research is that health care professionals’ concerns about their autonomy, the relationship between patient and the health care professionals related to using telehealth technologies [7, 8], and also health care professionals’ scepticism have been reported as possible barriers to implementation [9, 10]. Although one study of telemedicine within a health care district in Finland illuminated the importance of communication between health care professionals for the quality of patient care [11], the available information on broader issues, including inter-professional collaboration at the community level, is as yet quite limited [12].

Where follow up for people with diabetic foot ulcers has been studied, limited communication between their specialist and primary health care services has been reported [13]. More evidence is needed to better plan future telemedicine interventions aimed at improving such communications. Introducing new technology, such as telemedicine, in already existing practices is recognized as a predictable challenge for health care professionals [11, 14]. To obtain knowledge of their experiences early in the process may give us insights into how it is to use this new technology and what kind of structures could be seen as obstacles and facilitators to uptake.

The aim of our study was therefore to explore health care professionals’ experience in the initial phase of introducing telemedicine technology in caring for people with diabetic foot ulcers. By focusing on integrated care, this study is intended to contribute to our capacity to more comprehensively describe the processes taking place in complex interventions. This knowledge may help us in understanding factors which might facilitate or impede the introduction of telemedicine service delivery. As telemedicine is considered a potentially viable approach to optimizing health care delivery in many areas of chronic care [8, 15], such insights may have relevance beyond the delivery of diabetes foot care.

## Methods

We used an inductive Interpretive Description (ID) approach for this study [16, 17]. Informed by design technique developed for the purpose of social science methods such as grounded theory, phenomenology and ethnography, ID is a strategy for optimizing qualitatively derived knowledge development for use in the applied clinical fields [18]. A hallmark of ID is its recognition that the applied fields require moving beyond description of phenomena to search for interpretive understandings and meanings expressed in a form that is amenable to being put to use by the intended audience [16].

### Participants

The participants were recruited from the intervention arm in the ongoing cluster randomized controlled trial (RCT) (Clinicaltrials.gov: NCT01710774). Purposive sampling among health care professionals in this intervention was used to ensure that the sample included participants from home based care, a nurse-led primary care clinic, a medical center and outpatient hospital clinics. Invitations to health care professionals to participate in focus groups were issued by e-mail. Participants from seven home based care services, one nurse-led primary care clinic, one medical center and two outpatient hospital clinics ultimately attended the focus groups. Most participants were nurses (*n* = 29), but the sample also included one nurse assistant, podiatrists (*n* = 2) and physicians (*n* = 2). Six nurses, one physician and one podiatrist attended in two groups, the second one year after the first one. Originally, the plan was to interview all participants twice to see if the experience changed over time. Since relatively few patients fulfilled the inclusion criteria, the health providers recruited in the first round did not get appreciably more experience after one year. Hence, we decided to invite new participants into the second round of interviews.

The participants had worked as health care professionals from two to thirty seven years, and eighteen of them had completed advanced nurse education such as wound care, diabetes-nurse specialist or intensive care nursing education. Their work experience with wound care ranged from one year to thirty years. The participant’s mean age was 47 years (range 24–64), and only one man participated.

Although the participants had experience working with diabetic foot ulcers, they were all in the initial stages of introducing telemedicine in their work as a result of their participation in the ongoing cluster RCT. Those working in specialist health care were more experienced in using telemedicine than those working in primary health care.

### Data collection

We used focus groups for collecting data. Focus groups are suitable when the goal is to uncover both commonalities and variation in experiences of a shared situation or type of practice [19].

We conducted ten focus groups. The focus group interviews were conducted during 2014 and 2015. First author BCHK was moderator and EG was co-moderator. We chose not to mix participants from primary health care and specialist health care due to a possible difference in experience in using telemedicine, assuming that this could make some of the participants reluctant to talk in a group. We considered that mixing different health care professions within their own working context was appropriate so that different perspectives within their context could be explored and discussed.

The focus group interviews lasted from 70 to 90 min and were audiotaped. We used a semi-structured interview guide which covered topics related to our study aim. The main topics addressed are listed in Table 1. During the interviews the participants were encouraged to raise other issues that they considered important to bring forward.

**Table 1:** Topics in the interview guide

• Participants’ experiences in using telemedicine, and how it was organized where they work • Participants’ experiences in using telemedicine as a new tool in documentation and communication • Participants’ experiences in communication and collaboration between outpatient clinic (physicians, nurses and foot therapists) and nurses in home care through tele-communication, and among professions • Changes in their competence in caring for people with diabetes foot ulcers during the intervention • Changes in their job satisfaction while using telemedicine	

### Analysis

We used the ID approach in the following way:

Step 1: We started by a short debriefing right after each interview. Notes were taken during the interview by the co-moderator and these helped us in the analysis process by focusing our attention on particular issues that were raised by the participants. All interviews were transcribed verbatim by the first author. The first author read the transcripts repeatedly to become familiar with the data, and also wrote down initial topics arising from the verbal material as well as other ideas and analytic curiosity that attracted her attention.

In keeping with the intent to approach this topic with an open mind, the development of the semi-structured interview guide created an opportunity to reflect on the possible implications of prior conceptions that might influence the data generated through the focus groups. The telemedicine program to be evaluated in this study represents a new way of delivering a health care service for the participants. As a registered nurse and a diabetes specialist nurse with extensive knowledge of the health care system, the first author had some expectations in terms of the kinds of barriers one could face when introducing a new tool in diabetes care. We tried to be aware of such expectations and the implications our various preconceptions may have had on the data constructing during the analysis phase.

Step 2: We started out with three of the authors reading all the transcripts. The first author started the initial coding process using an open-ended, inductive approach (i.e. seeking to avoid granting the pre-existing themes from the semi-structured interview guide too much attention). A constant comparison method was used to compare the different pieces and patterns in each interview. Open coding was used to capture issues and ideas in the data. The process was done by coding terms that were as broad-based as possible to avoid premature closure on interpretation in accordance with the ID approach. Coding was also done independently by EG and MK, and all authors discussed the coding during and after this process to uncover the most important issues in the data.

Step 3: After this process the authors also took into consideration the explicit aims of the study, paying attention to whether the initial coding might have been different had the semi-structured interview guide been kept in mind. Considering the data set from multiple angles allowed for consideration of various patterns and differences across these interviews and possible relationship between these patterns were discussed by all authors. We concluded the analytic process by summarizing aspects which most fully captured issues of thematic importance in the data.

### Ethical considerations

The study was approved by the Regional Committee for Medical Research Ethics (2011/1609/REK vest). The participants were informed that their participation was voluntary, and that it was fully possible to withdraw from the study at any time with no consequences. Their anonymity was preserved by using numbers instead of names in all transcripts.

## Results

We were surprised by how overwhelmingly positive and enthusiastic the participants were with regards to using telemedicine, and how well it was received. We came to understand that there was a generally held view in all of the focus groups that the participants experienced meaningful changes, and the changes had benefitted their practice in three ways:

1. Increased wound assessment knowledge and skills
2. Improved documentation quality
3. Streamlined communication between primary health care and specialist health care.

### Increased wound assessment knowledge and skills

The health care professionals found that they increased their wound assessments skills when using telemedicine in their follow-up care for people with diabetic foot ulcers. The interactive web based ulcer record supported their systematic wound assessment, helping them in what to consider as relevant in wound care, and the use of this web based ulcer record facilitated an increased dialogue and reflection around wound treatment among health care professionals. They held the belief that, as a result of the intervention, they were better at seeing factors around the patient which might affect the wound and the treatment, such as the diabetes disease, nutrition and exercise. Especially they found the web based ulcer record important in this regard:

*“We have increased our wound assessment skills by using this web based ulcer record. And we have much more competence in looking at the person with a diabetic foot ulcer in a holistic way.”* Similar statements were expressed by nurses both in primary health care and specialist health care.

However, the significance of the web based ulcer record varied depending on where they worked. Those working in primary health care were more aware of using the written documentation and images to increase their skills than the health care professionals in the outpatient hospital clinics:

*“We learned to assess a wound when reading the documentation from the outpatient hospital clinic. Their notes help us.”*

Even though nurses in primary health care were quite certain that their competence had improved because of using telemedicine, they held forward the possibility that this also could be an effect of the fact that many of them had completed advanced education in wound care recently. As one of the nurses in primary health care said:

“I am not sure if it’s using telemedicine or that I have completed my education in wound care that raised my competence level.”

On the other hand, the nurses working in the outpatient hospital clinics experienced the web based ulcer record as easing their job related to when they should advise their primary health care colleagues or adjust the treatment being delivered in primary health care settings by others. The use of images combined with text made the clinical issues much more visible and therefore understandable to them all. They had much to say about passing on knowledge to those working in primary health care by using this web based ulcer record. Some examples illustrate. As one specialist health care nurse explained:

*“Maybe they can learn from our wound assessments that there are other things as well, which should be considered in wound treatment. This web based ulcer record forces health care professionals in home based care to see that it’s not only a wound to focus on, but also the complexity of it all.”* A nurse from the outpatient hospital clinic nodded eagerly and said: *“We can challenge them to do their own wound assessments, to which we can then give them some feedback on. I’m sure that it would help them to become safer and more interested in this field of nursing.”* This competence aspect led to a discussion within the specialist health care focus groups, where the opinion varied. One nurse said: *“I believe that there are many health care professionals in primary health care with a lot of competence, and maybe they don’t agree with us upon the treatment. This can lead to many discussions between us which are important. Even though we work in the specialist health care it doesn’t mean that we always know best.”*

This concurs with opinions expressed by nurses in the primary health care. They also wanted their colleagues in outpatient hospital clinics to recognize their competence, and they certainly would like to be asked for advice on wound care treatment.

The primary health care participants in particular, experienced that using images as well as electronic text in the web based ulcer record facilitated increased dialogue and more reflection around wound treatment between colleagues. This aspect was spoken about frequently in the focus groups, and can be summarized as: *“I find it easier to discuss with my colleagues when we can look at the same picture back at the office.”* They expressed that they developed a mutual clinical language with the specialist health care when using telemedicine. This seemed to enhance their wound assessment skills. It also seemed to those involved in telemedicine that the level of interest in the work of foot ulcers of other colleagues not involved in the intervention was increasing: *“They are more interested now, because of the images I have showed them, they have asked more and have been much more eager to learn more about wound treatment.”*

Using images and words together in the documentation gave the health care professionals a tool which enabled them to discuss and reflect upon wound treatment with other colleagues who hadn’t seen a particular diabetic foot ulcer previously. Discussion and reflection with colleagues led to an increased interest in diabetic foot ulcer treatment and learning new skills, both of which also spilled over to colleagues not involved in the intervention. On the other hand, health care professionals working in outpatient hospital clinics did not find this to represent a significant change in practice. They were accustomed to discussing such issues with one another more often during the day.

The participants in primary health care and specialist health care experienced increased confidence as health care professionals with better skills in wound assessment. This increased confidence made them more accurate and clear in the language they used in discussions with other health care professionals. They dared to disagree and keep on with the discussion when it was important. All of this made them a more competent health care professional in their own eyes. This new confidence seemed to enhance what had already seemed a high level of job satisfaction.

### Improved documentation quality

The interactive telemedicine platform used by primary health care and the outpatient hospital clinics also seemed to facilitate a process where the health care professionals gradually improved the quality of their documentation. The web based ulcer record required that health care professionals follow the same standards in reporting their findings. This feature seemed to direct attention to aspects of primary concern in the web based ulcer record. It was commented upon in this way in some focus groups in outpatient hospital clinics:

*“…with this new tool we use now, it is easier to systematize and map our wound assessments. I think we are….when I look at our notes I find that we are very much to the point.”*

It was a generally held view both in primary health care and outpatient hospital clinics that this experienced quality improvement in documentation was significant to them. As one nurse in primary health care stated: *“I think we are better able to document the essence now, and it’s much more straightforward and factual now.”* Another responded with: *“We are more to the point in our documentation now, and we are better in seeing the whole person.”*

Using this interactive web based ulcer record with documentation in both words and images enabled them to work more systematically when documenting and when giving practical care. The documentation became more precise both in structure and content as a result of the technological innovation. The high level of enthusiasm outpatient hospital clinic professionals had about this aspect was apparent in the lively nature of their dialogue in several of the focus groups. As one commented: *“The notes are quite different after we started using telemedicine. There is much more information in our documentation now, also because we use images.”* Similarly, one doctor said: *“Actually the quality of our notes is better, also because we use images as well in our documentation.”*

We identified several factors that facilitated and inhibited the use of telemedicine, and some of those may explain why the health care professionals experienced this improved quality in documentation. First of all, the health care professionals involved found the telemedicine equipment and the web based ulcer record easy to learn and use. Few technological problems occurred during this initial phase. Furthermore, the participants received technical support quickly from more experienced people at the outpatient specialist health care setting. Many of them had completed advanced courses during recent years in wound treatment, and were highly motivated and enthusiastic when starting to use telemedicine. This together with being followed up by specialists when they were using this wound journal made them more confident in what to document. The participants also experienced that their suggestions for improvements on the technology were taken into account and improvements were made.

On the other hand it was a generally held view that the documentation process was too time consuming because they had to do double documentation. This was due to journal systems which were not integrated with each other. In primary health care some participants were told by the management not to use much time in documenting at all because it would take time from other tasks. They also experienced that other colleagues commented negatively on time spent with documentation and tasks related to telemedicine. Some of them expressed that documenting in the web based ulcer record and using the telemedicine equipment gave them more responsibility than the rest of the staff and not everyone liked that.

### Streamlined communication between primary health care and specialist health care

The final main benefit that the health care professionals spoke of was the intervention’s effect on streamlining communication between their two sectors. In most of the non- specialist settings that these professionals represented, one or two selected nurses were assigned the responsibility for using the telemedicine equipment to transfer images of the foot ulcer captured by mobile telephone for assessment. These nurses could then perform wound care in cooperation with the specialist health care staff.

The participants in both primary health care and specialist health care experienced that this contributed to streamlining the communication between them. Their dialogue brought forth recollections of a lack of communication between them prior to the telemedicine intervention. As two of them commented, *“We communicate more with each other now. We hardly did that before”* and *“Yes, we have much more contact now when using telemedicine.”* Interestingly, their accounts demonstrated some variety in terms of the extent of their communication prior to telemedicine. For example, in one focus group in primary health care, one participant explained that, “*Our communication with specialist health care was very good earlier and still is. There have been few changes regarding communication.”* Another quickly commented, “*We had no contact with the specialist health care, so this is new to us.”* Regardless of this diversity the increased contact seemed to make the communication more efficient in terms of being timesaving and conducting better treatment and care.

As an outcome of this expanded communication occasioned by being introduced to the other people through telemedicine, participants explained that it was easier for them to reach the right person on the other end when they wanted to discuss or pass along messages about a case. They appreciated knowing who to contact when needed and it made it more personal to them all. They believed this way of communicating lowered the threshold to initiate contact between health care levels. *“Now, we don’t have to do all those phone calls, just to find the right person”* and *“We find it very timesaving”* were remarks indicative of what many seemed to be feeling in both health care levels involved.

Health care professionals in primary health care seemed to be very clear and satisfied by the fact that they didn’t have to communicate with the patient’s GP for advice in wound care anymore. Although some were also concerned about the GP’s need for being updated on the wound care, they found it easier to make contact directly with the outpatient clinic when needed. A comment like this was common: *“It’s great to have the opportunity to communicate with the outpatient clinic instead of trying to reach the GP, who delays us.”*

One primary health care nurse expressed surprise at how easy it had become to reach out for specialist advice: *“It’s very easy to make contact with specialist health care, there is always someone to talk to.”* Another contrasted the open attitude she experienced in such consultations with her prior assumptions about the specialist sector: *“We experience specialist health care to be very helpful. We are not used to that.”*

The value of this streamlined form of communication for the effectiveness of care as well as their satisfaction with providing it was captured in one primary care nurse’s summary: *“This communication makes us more confident on the fact that ongoing treatment and adjustments are followed*.”

Thus, in these three respects – wound assessment, documentation, and communication - practitioners saw enhancements in their practice and developed a sense of confidence that patient care was ultimately enhanced by the introduction of telemedicine.

## Discussion

Our findings showed that introducing telemedicine in primary health care and specialist health care revealed a change in wound assessment knowledge and skills. Images combined with text facilitated the process. Images were the key element, but written text in the web based ulcer record was also vital to further understand the image better. They developed an increased understanding of what they actually saw when looking at the foot ulcer when doing wound care and when reporting. Images were seen as more informative than text, and were an essential part of the learning process. Communication between health care professionals when using images enabled them to work in the same context: their references were the same. Having the same patient and image to focus on placed their wound assessment in the same context, which seemed to make a difference in a learning process. Through the dialogue between primary health care and specialist health care they became more conscious and reflected on their work and this developed their competence. The importance of using images in wound care was also advocated in an American study [20] where the author found that not using digital images in wound care in nurse-to-nurse consultations might lead to under - or over-treating patients’ wounds. They also saw that using images enhanced a more holistic assessment of the patient.

Information and communication technologies may also provide those using it with an innovative learning situation such that stimulation and learning processes are enhanced, also seen in a Spanish study with students and general influences on student learning [21]. Further, enhanced competence when using electronic devices while communicating with other health care professionals has also been found in other studies of health care professionals’ experiences when using electronic devices and telehealth [11, 22, 23]. In our study the health care professionals in primary health care experienced a development of a professional wound care language which enabled them to participate in discussions with colleagues in a more confident way. Using the same clinical language seemed to enhance their learning when assessing a wound and contributed to practical skills in wound care. Their increased wound assessment knowledge and skills when using telemedicine seemed to facilitate their confidence in their own skills, and they learned and grew as health care professionals. Similar findings were seen in Danish and Finnish studies [11, 24]. These studies found that competence exchange by using electronic devices could increase the nurse’s competence.

Change in technology or using new technology impels change in practice. It has been suggested that using this new technology might lead to “tunnel vision” or becoming preoccupied with the technology [25]. Interestingly, in our study health care professionals did not report any narrowing of perspective. On the contrary, they explained that the technology helped them actually to see the patient in a more holistic way as the new technology stimulated a broader assessment of the patient’s situation. Using the web based ulcer record seemed to facilitate a process of increased dialogue and reflection where the participants gained more competence in understanding diabetic foot ulcer and knowing what to consider and report around the diabetes disease. In this way, using this web based ulcer record seemed to provide them with a tool which helped them see a larger bit of the whole picture. Similar findings are reported in other studies [11, 24].

The positive response to using information and communication technology that we noted in the participants of this study is also reported in some other studies which consider health care professionals’ experience when using new technology [1, 22, 25–27]. The documentation aspect in our study was seen as time consuming partly because of the double documentation they had to do. Ongoing development in the technology might reduce time spent on documentation, and this seems important to prevent frustration that may discourage the adoption of this new technology. Nevertheless, it didn’t change their enthusiasm. This might be a result of the good quality of the telemedicine equipment and the follow-up support when using it. It was easy to learn and to use. They experienced the possibility of influencing the design of the technology application when needed, and this is in accordance with a Swedish study [25] where health care professionals reported the importance of being able to influence in the introduction of this new technology. Having equipment which is easy to learn and use, and being able to trust the technology together with a good technical support is important for health care professionals using telehealth technologies [7, 28]. As has been reported in other studies [7], avoiding these barriers seems a crucial consideration when adopting telemedicine.

The participants in both health care levels experienced that the web based ulcer record functioned as a kind of guideline in wound care treatment because the web based ulcer record was specific in what to document. This led to increased quality of their written documentation. The follow-up by specialists when using this web based ulcer record made the participants in primary health care more confident in what to document and created a positive learning spiral. Increased confidence is also noted in the literature [24]. This follow-up from specialist health care seemed to be crucial for their satisfaction and acceptance of the intervention when using telemedicine, an element that has also been noted by others [7]. It seems that the telemedicine system, and the web based ulcer record in particular, complemented their existing knowledge and supported them in decision making by giving them confidence and security in practice. This element of the positive effects of working with guidance from experts as well as obtaining support from the system has also been reported in other studies [23, 24]. The findings in this study may help improve deliverability in other telemedicine interventions by focusing on how healthcare professionals’ communication, contact and support progress between colleagues and across levels of health care.

One could ask why the health care professionals were so enthusiastic when being introduced to telemedicine. One of the explanations for this could be the fact that there has been little communication between primary health care and specialist health care in this context prior to the introduction of telemedicine. The lack of communication and coordination between primary health care and specialist health care in a more general sense has also been a main concern for politicians in Norway [29]. The new informal communication between health care levels when using telemedicine was valued very highly by those involved in our study. This communication made it easier to make ongoing adjustments and plans for further treatments. This form of contact between sectors was something new, predictable and believed to be ultimately very timesaving by all the health care professionals. Thus, telemedicine may be one means to bridge the gap between the primary health care and specialist health care.

### Implications for practice

Our findings shed light on the process health care professionals experienced taking part in a telemedicine intervention. Our findings highlight the need for tailoring an intervention to the context in which it will take place, and insights from the health care professionals’ experiences might help us this regard. Clearly, if telemedicine is to become more widely implemented it will be important to give practical training and education about it to all health care professionals in primary health care, not simply to a few.

### Methodological considerations

Our findings should be seen as a contribution to gain a better understanding on how it is for health care professionals to start using new technology in an already established working practice. The findings represent one angle of vision and cannot automatically be transferred to other settings and patient groups. Nevertheless we consider them to have potentially important implications for using telemedicine across levels of care. A strength in this study is that the participants varied in their place of work, working experience with foot ulcers, their age and their profession, thereby proving a wide variety of experiences and perspectives on the use of telemedicine. Those working in specialist health care were more experienced than those working in primary health care, but all participants were in the initial stages of using telemedicine and thereby provided useful insights to consider when launching new technology.

## Conclusions

Our findings indicate that using a telemedicine intervention enabled the participating health care professionals to approach their patients with diabetic foot ulcer with more knowledge, better wound assessment skills and heightened confidence. They found telemedicine to streamline the communication between health care levels and see their patients in a more holistic way. Furthermore, our findings also demonstrate that participants involved in telemedicine intervention can develop as health care professionals. Thus, we see considerable promise in the ongoing exploration of the experiential dimension of communication technologies as they continue to unfold in the evolution of health care systems.

### Ethical approval

Western Norway Regional Committee for Medical and Health Research Ethics approved this study (Project no: 2011/1609/REK vest).

### Availability of data

Western Norway Regional Committee for Medical and Health Research Ethics approved this study. Due to ethical and legal restrictions related to confidentiality, the data cannot be deposited online as the study participants have not explicitly been informed about, nor approved data sharing when the data were gathered in 2014–2015.
